# A novel quantum algorithm for efficient attractor search in gene regulatory networks

**DOI:** 10.1016/j.patter.2025.101295

**Published:** 2025-07-03

**Authors:** Mirko Rossini, Felix M. Weidner, Joachim Ankerhold, Hans A. Kestler

**Affiliations:** 1 Institute for Complex Quantum Systems, Ulm University, 89069 Ulm, Germany; 2 Center for Integrated Quantum Science and Technology (IQST), Ulm-Stuttgart, Germany; 3 Leibniz Institute on Aging, Fritz Lipmann Institute, 07745 Jena, Germany; 4 Institute of Medical Systems Biology, Ulm University, 89069 Ulm, Germany

**Keywords:** quantum computing, Boolean networks, gene regulatory networks, quantum amplitude suppression, attractor search

## Abstract

Describing gene interactions in cells is challenging due to their complexity and the limited microscopic detail available. Boolean networks offer a powerful, coarse-grained approach to modeling these dynamics using binary agents and their interactions. In this context, attractors—stable states of the system—are associated with biological phenotypes, making their identification biologically important. However, traditional computing struggles with the exponential growth of the state space in such models. Here, we present a novel quantum search algorithm for identifying attractors in synchronous Boolean networks, specifically designed for use on quantum computers. The algorithm iteratively suppresses known attractor basins, increasing the probability of detecting new ones. Unlike classical methods, it guarantees the discovery of a new attractor in each run. Early tests demonstrate strong resilience to noise on current NISQ (noisy intermediate-scale quantum) devices, marking a promising advance toward practical quantum-enhanced biological modeling.

## Highlights

• A novel quantum algorithm for attractor search in Boolean networks and GRNs
• Each attractor found in one run using quantum amplitude suppression
• The method shows robustness to noise on current quantum hardware
• Bridges genetic modeling with quantum computing methodologies

## The bigger picture

As classical computing approaches physical and practical limits in solving combinatorial problems, quantum computing offers a promising alternative by exploiting principles such as superposition and entanglement. These features can also prove valuable in fields such as systems biology, where analyzing gene regulatory networks (GRNs) involves inherently complex and high-dimensional dynamics. Boolean networks, widely used to model GRNs, face scalability issues when applied to exhaustive attractor search—a task central to understanding stable gene expression patterns and cellular phenotypes. This work presents a quantum algorithm designed to find all attractors in synchronous Boolean networks with improved efficiency, offering computational advantages, particularly in biologically relevant networks that operate near criticality and often exhibit complex landscapes with a limited number of critical attractors. This proof of concept builds a methodological bridge between quantum computing and gene regulation modeling, providing a new tool for exploring biological complexity with quantum-native approaches.

In the future, this strategy can be expanded to larger biological systems at the hardware scale and seamlessly integrated into hybrid classical-quantum pipelines. This sets the stage for quantum computing to be utilized not merely as a theoretical novelty but as a functional component of computational biology and bioinformatics.

## Teaser

Gene regulatory networks (GRNs) are a fundamental framework for studying cellular behavior. They can be modeled as Boolean dynamical networks, but their complete characterization is often computationally intractable. This work presents a quantum algorithm that efficiently identifies all attractors of such networks using iterative quantum amplitude suppression, achieving performance improvements over classical approaches. The method is validated on quantum simulators and demonstrates robustness to noise, making it a promising candidate for current and near-term quantum computing applications in biology.

## Introduction

DNA encodes the essential information for constructing all cellular life on Earth. Its information is translated into the synthesis of proteins, which serve as tools for a variety of intracellular tasks. One very important one is the regulation of the translation process as such (gene expression). While urgently needed, the immense number of agents and the lack of knowledge about their cooperation render a microscopic description of gene regulation unfeasible. However, in the last decades, driven by advances in genetic technologies and computational power, it has turned out that in many cases, coarse-grained effective modeling is sufficient and powerful enough to examine crucial aspects of, for example, entire networks of genes, mRNAs, and proteins, as well as to provide detailed descriptions of, for example, signaling cascades and crosstalk between pathways.^1^

In general, networks consisting of genes and their mutual interactions are called gene regulatory networks (GRNs). These networks aim to capture the most relevant mechanisms for the self-regulation of the cell’s metabolism. Advanced numerical methods have been developed to tackle their complexity; among many, ranging from differential equations^2^ to methods based on Petri nets,^3^ Boolean networks (BNs) are considered one of the most powerful ones. They are able to capture essential phenomena observed in experiments, can easily be adapted to include emerging additional information, and allow us to make accurate predictions about the behavior of a system and, i.e., more generally, its phenotype.^4^

BNs have already been investigated for the analysis of a wide range of systems, including development,^5^ cell cycle regulation,^6^ hematopoietic stem cells,^7^ and cancer.^8^^,^^9^ In particular, the problem of attractor search in BNs, which we define mathematically later in this manuscript, is a fundamental task in the field of system biology, as these specific states of the system are often related to the genetic expression and phenotype of the cell.^10^ The most challenging problem is identifying all attractor states by exhaustive system dynamics simulation. This requires the computation of the full state transition graph (STG), a directional graph consisting of all possible $N=2^{n}$ configurations for the Boolean activity of n genes, and its evolution in time (via directional links), thus making it an NP-hard problem.^11^^,^^12^^,^^13^

Interestingly, the benefits of gaining insight into the dynamics and properties of BNs are not limited to systems biology but extend to many-body physics. Namely, such a framework can be considered as a generalization of the famous Ising model, a chain of interacting spin-1/2 encoding the states “up” and “down.” The transverse Ising model is currently being investigated for its potential applications as a quantum simulation platform. Recently, several experimental realizations have been explored, including artificial spin ice (ASI) arrays,^14^ trapped atomic ions (Kim et al.^15^ and therein), neutral atoms,^16^ and superconducting circuits.^17^

One may thus wonder whether the challenge of identifying attractor states in GRNs can be tackled with tools from quantum information processing. The answer is indeed affirmative and is precisely what we provide here. We present a novel quantum algorithm that lays the basis for a fundamental boost in tackling even rather complex GRNs of high biological relevance. Indeed, we prove that it is possible to design interference properties of quantum devices to suppress any basin of attraction of one or more previously found attractors. This ensures that different subsequent runs of this algorithm identify a different attractor at each different run, thus achieving the highest level of optimization possible for a classically inspired search method based on multiple simulations and measurements of a state after it has evolved in time.

We prove the efficiency of our method on two model BNs, one created ad hoc and the other taken from the literature.^5^ By running our algorithm on a quantum computer simulator, we show the exactness of the results for the problem at hand. We then run the algorithm on a quantum computer simulator capable of mimicking the noise profile of a target quantum device (provided by IBM and the Qiskit Python package) to show the effect of noise on the final results. These results convincingly demonstrate that the algorithm provides solid and reliable results even on today’s quantum devices (specifically the *ibm_brisbane* processor), regardless of their nature as noisy intermediate-scale quantum (NISQ) devices. These results are complemented by a detailed algorithm breakdown, both in its circuitry and mathematical formulation.

## Results

A method that has proven extremely efficient for characterizing the dynamics of GRNs is to map them onto a dynamical network of Boolean variables, i.e., a BN. Characterizing the properties of such maps is therefore crucial for uncovering the molecular mechanisms behind the phenotypic expression of a cell, including genetic patterns that lead to pathological states, such as cancerous behavior or genetic diseases. Because genetic interactions in cells occur on a very fast timescale, not all possible states that the BN can assume are relevant to the phenotypic expression of a cell, only those that are stable and do not change over time. Such states are called attractors of the BN. Different schemes can be used to model the time evolution of such networks, such as synchronous update schemes (all variables are updated at the same time), asynchronous update schemes (different variables can be updated at different times), and more.^4^ In this work, we focus on synchronous BNs.

Many different classical methods have been developed to obtain more or less accurate results for the problem of determining the attractors of a BN, taking advantage of the better efficiency of algorithms that require less precision. In this work, we prove an algorithm for quantum computation that, by representing the problem in terms of qubits and quantum gates, manages to achieve better efficiency in solving the problem exactly, i.e., by finding each and only the attractors present in a general synchronous BN without loss of accuracy.

Given an ensemble of n Boolean variables (each representing, for example, a gene of the GRN), $2^{n}$ possible states for the network always exist. A set of rules (Boolean functions [BFs]) can then be used to determine their dynamics over time, i.e., how a given state of the system is repeatedly mapped to a new one (possibly itself again) at each time step. The collection of all $2^{n}$ possible system states and their dynamical evolution is called an STG.^4^ As mentioned in the introduction, the exhaustive computation of such a graph scales exponentially. Thus, it can be proven that the problem of finding both single-state and multi-state attractors in BNs is NP hard.^11^^,^^12^^,^^13^ This limits exhaustive simulations to small networks, e.g., using the BoolNet package in R,^18^ to n≤29. For larger systems, heuristics are used that can guarantee finding the entire attractor set but do not provide any additional information about the attractor basins. Such heuristics can be based on Boolean satisfiability (SAT)^19^^,^^20^ or equivalent methods.

The quantum approach is inspired by its classical counterpart but exploits the possibilities offered by quantum superposition and quantum interference of qubit states. It can be divided into two main parts, the first being subdivided into 5 steps (a detailed breakdown of the algorithm is provided later in algorithm description). The first part consists of initializing the qubit system to the desired initial state. Such a state, in the first execution of the algorithm on the quantum device (from now on called runs of the algorithm), is the superposition of all possible $2^{n}$ states that the system can be in. As often done in the literature, it can be obtained by applying a Hadamard gate to each qubit. Then, remembering that each possible state of the network represents an orthogonal dimension in the Hilbert space of all the qubits, in the second part, we apply the BFs to such a prepared state, thus evolving each state belonging to the superposition into each time-evolved state. In the first run, by evolving the initial Hadamard state a sufficient number of times, we will eventually recover a superposition of all and only the static attractors of the network and elements of the dynamical attractors. The measurement of such a state would now result in one of the attractors of the system (or a state belonging to a dynamical attractor). In their recent work, Weidner et al.^21^ successfully implement such BFs on a quantum device and couple them with a Grover search algorithm^22^ to efficiently explore the basins of attraction of the attractors of a network, provided, however, that such attractors are known in advance. A more detailed methodology description can be found in Weidner et al.^23^

The next step is, therefore, to be able to efficiently measure all the different attractors of a given network while avoiding measurements of the same result multiple times (e.g., by measuring an attractor of the network with a particularly large basin of attraction multiple times, as in classical methods), thus optimizing the algorithm’s efficiency. So, once the first attractor of the system has been measured, in order to measure the next attractor, we will again apply the two steps described above, this time defining a different strategy for initializing the qubit system at the beginning. In fact, we want to develop a state preparation strategy capable of preparing the initial state in the superposition of all the possible states of the system *except* the states belonging to the basin of attraction of the first attractor found. In this way, we can ensure that our subsequent measurement after applying the BFs to the system will uncover a different attractor than the one initially found. To this end, we have developed a novel algorithm that applies an optimized amplitude-suppression strategy to this problem inspired by the principles of the Grover algorithm. The details of our development of this algorithm can be found in algorithm description below. The iterative application of this method to any BN would allow a different attractor to be found each time the algorithm is run, ensuring that all the system’s attractors are found within a number of runs equal to the number of attractors present in the network.

While our algorithm efficiently identifies all attractors in a BN within a number of runs equivalent to the number of attractors themselves, it is not optimal for all network configurations. In pathological scenarios—such as networks with a state-space structure forming long linear chains (e.g., each state having an in-degree of one except a single state) or networks with a very large number of attractors (up to $2^{n}$, i.e., each state is an attractor)—the required number of qubits and/or the circuit depth would increase, limiting the application of our quantum method in current NISQ devices, and classical methods could prove more practical. For example, after a transient time (number of time steps) $T_{t}$, the probability of finding an attractor in the first pathological case presented above would be $P_{att}=\frac{T_{t}+1}{2^{n}}$. Thus, a preliminary structural and complexity analysis of the network aimed at investigating, for example, the state-space diameter^24^ or complexity metrics, such as basin entropy,^25^^,^^26^ can greatly benefit researchers by indicating whether a classical or quantum approach is best suited. On the other hand, our quantum method proves particularly advantageous for scenarios where attractors with significant basin sizes are partially characterized, and the challenge resides in identifying the remaining elusive attractors. Moreover, it proves useful for biologically inspired BNs (e.g., GRNs), as such networks frequently exhibit canalized regulatory logic and operate near criticality, characteristics usually associated with a polynomial scaling number of attractors in the network size and relatively faster system convergence,^27^ which aligns well with the strengths of our quantum approach.

Finally, we would like to point out that this method works exactly not only for so-called single-state attractors (single states in which the system remains once reached) but also for identifying multi-state cyclic attractors of a network (set of states through which the system repeatedly cycles). In the latter case, our algorithm would correctly identify a state belonging to the set of dynamic attractors. Uncovering the whole cycle is a trivial task that can be done later with a classical implementation of the Boolean state transitions that generate the GRN. An example of such a routine is presented in example run with cyclic attractors in the supplemental information.

### Examples

**Figure 1:**
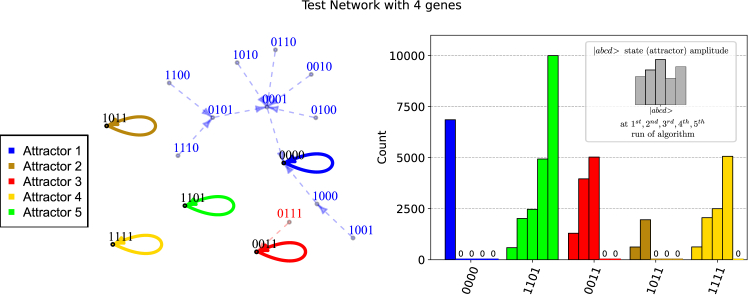
Quantum algorithm run on a 4-gene test network On the left: schematic representation of the Boolean network generated by 4 interacting agents designed ad hoc as a test case (see supplemental information for further details). The attractors are labeled with the order of the experimental measurement in our example. On the right: count probability of measuring each system attractor after each algorithm run. The attractors found in the previous run are suppressed in the following runs. For clarity, the histogram highlights “0 counts” results.

**Figure 2:**
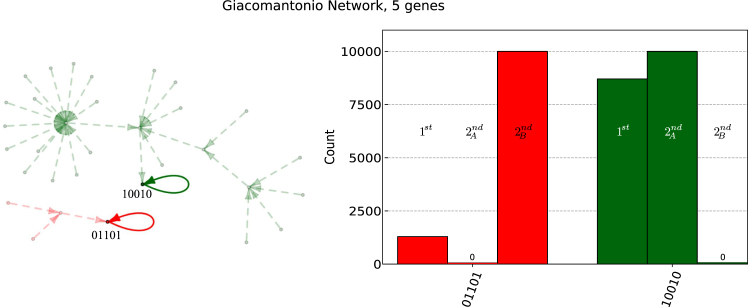
Quantum algorithm run on the 5-gene Giacomantonio network On the left: schematic representation of the Boolean network generated by 5 interacting genes described by Giacomantonio et al.^5^ Only the two attractors are marked for better readability. On the right: count probability of measuring each attractor of the system over a total of 10,000 runs. The first run $\left(1^{st}\right)$ describes the probability of measuring one of the two attractors in the first run of the algorithm. Run $2_{A}^{nd}$$\left(2_{B}^{nd}\right)$ describes the probability of measuring the green attractor (red) after measuring the red attractor (green) in the first run using our algorithm. For clarity, the histogram highlights “0 counts” results.

We prove our results on two networks, one with four interacting agents created ad hoc (details on its structure and updating scheme are reported in four agents test Boolean network in the supplemental information) and a real one introduced by Giacomantonio et al.^5^ with a real interaction map of five Boolean agents describing mammalian cortical area development. In Figures 1 and 2, we show the networks and the probability histograms for each state to be measured after each run of the algorithm, removing at each run the previously found attractors. For the smaller network (Figure 1), we show the subsequent removal of attractors in an arbitrarily chosen sequence. For the Giacomantonio network (Figure 2), which features only two attractors, we alternatively show the removal of one or the other attractor from the superposition, showing how the algorithm exactly removes the respective basins of attraction regardless of their sizes (4 or 28 out of 32 states). All results are performed on IBM Qiskit quantum device simulators. This allows us to demonstrate the determinism and exactness of the algorithm we present and the low impact of shot noise on the expected results.

The examples shown demonstrate the potential of the proposed algorithm on two different toy models, as quantum platform simulators are not equipped to simulate larger scenarios. Nevertheless, the number of qubits required by the algorithm increases with a scaling that is linear both in the number of Boolean agents and in the number $T_{t}$ of time steps required for the system to converge to its attractors, which often scales polynomially in the number n of agents for genetically relevant or critical networks (often $n^{0.5}$ to n).^25^^,^^28^ Models currently studied in the literature^7^^,^^8^^,^^29^ often have networks of about 40–50 genes and require about 10 time steps to converge to the attractors, implying a need for around 400 logical qubits to perform the analysis. The qubit overhead required to perform mild error mitigation techniques in NISQ devices—which the evaluations of our algorithm on noisy platforms provided in the next section estimate to be sufficient—requires the use of about 10 physical qubits per logical qubit. For this reason, it is currently not possible to solve classically infeasible networks using this method. On the other hand, recent developments by quantum platform manufacturers such as IBM and Google, together with their roadmaps for the coming years, show that the requirements estimated above may indeed be met within the next few years, making the use of this algorithm practical for state-of-the-art research purposes.

In the next section, we will present an analysis of the robustness of the algorithm against the noise present in currently available NISQ devices. Indeed, we can see in both cases how the impact of shot noise is not relevant to the scope of the algorithm and how the algorithm exactly removes the required attractors from the state superposition up to leaving only the last to be found, with 100% probability (10,000 shots).

### Noisy runs

**Figure 3:**
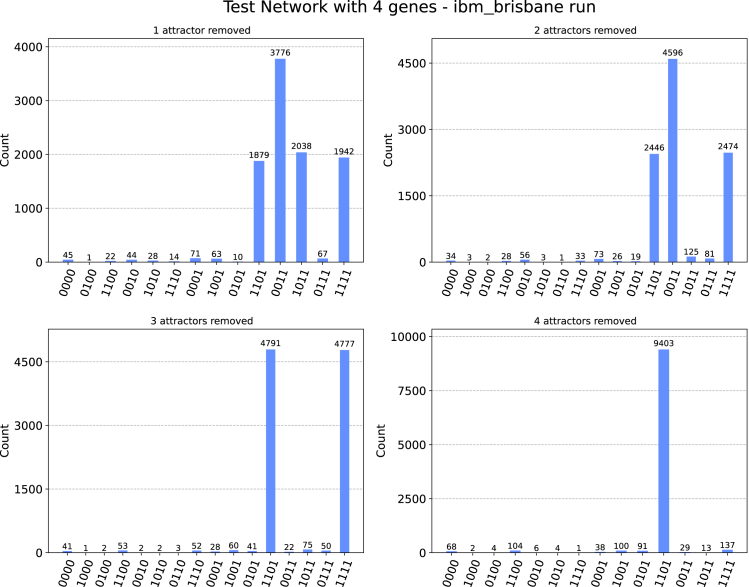
Subsequent runs of the deletion algorithm over the attractor basins of the 4-agent network introduced above We prove our algorithm on a simulator that considers the noise profile extrapolated by the *ibm_brisbane* quantum device. In each run, we delete one more attractor basin (assumed to have been measured in the previous run) in the order 0000→1011→0011→1111. The last one to be measured will be 1101. See the supplemental information for further details.

**Figure 4:**
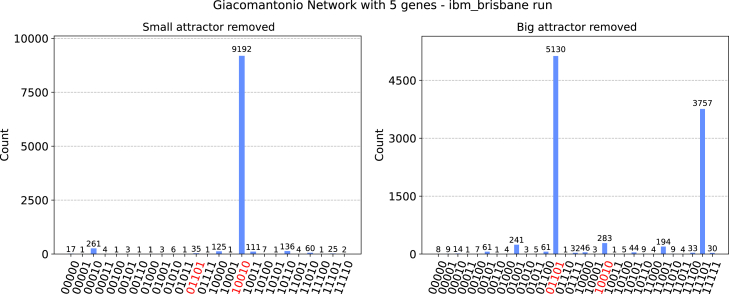
The deletion algorithm runs over the attractor basins of the Giacomantonio network introduced above We prove our algorithm on a simulator, taking into account the noise profile extrapolated by the *ibm_brisbane* quantum device. In these two runs, we alternatively suppress the basin of either the small (left) or the large (right) attractor (highlighted in red in both plots), allowing the measurement of the other.

The Qiskit package and IBMQ devices allow us to perform simulated runs of our quantum algorithm that mimic the error profile (the set of intrinsic probabilistic errors resulting from the imperfect NISQ nature of current quantum devices) of any machine currently available among the IBMQ quantum platforms. Since the choice of a particular platform among those presently available does not make a fundamental difference, we decided to use the noise profile of the *ibm_brisbane* platform. This allowed us to approximate the resistance of our algorithm to the noise generated by state-of-the-art quantum machines. The results are shown in Figures 3 and 4.

Figure 3 shows the same four runs that we previously showed in the “non-noisy” simulations in Figure 1. Each histogram represents one run as we remove the attractors one by one, assuming they are removed in the same order as before. If we define the error probability as the probability of measuring a state from a run that does not belong to the set of attractors yet to be found, the error probabilities for each of these four runs are, in order, 3.65%,4.84%,4.32%, and 5.97%.

In Figure 4, instead, we find the results for the Giacomantonio network. In the two plots, we can see the efficiency of the algorithm in removing both the small (left) and the large (right) attractor basins, thus allowing the measurement of the other attractor, assuming noisy conditions (*ibm_brisbane* noise simulator). Deleting the small attractor gives rise to the measurement of the big attractor with a 91.92% probability, with a noisy “grass-like” distribution of wrong outcomes filling the missing 8.08%.

Removing the large attractor basin to allow the measurement of the small attractor gives a slightly different result. While we still find the 51.30% probability of measuring the correct attractor and a grass-like distribution of small probabilities for most of the wrong results, we also still find a 37.57% probability of measuring a wrong result with a bit flip on the first qubit. However, such a situation can be easily overcome by introducing a hybrid quantum-classical routine, which checks the nature of each algorithm result with a classical computational algorithm. Checking the nature of a given state is a fast classical problem, which allows us to optimize the robustness of our algorithm against noisy results in a very efficient way.

Our results show how robust this algorithm is to the expected noise sources from the state-of-the-art quantum devices in the IBMQ fleet. Although the technical implementation of quantum computing on a large and ubiquitous scale still requires efforts from the scientific community, this tool proves to be a valuable and reliable technique to tackle a computational task widely used in bioinformatics, medicine, and many other fields.

### Algorithm description

**Figure 5:**
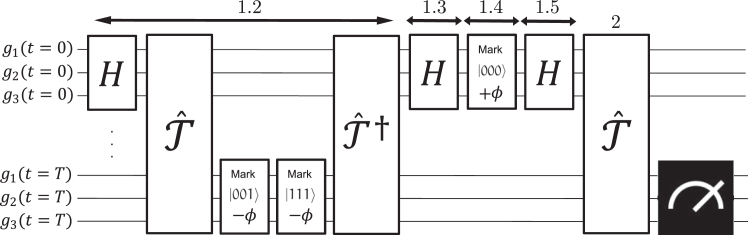
Schematic example of the basin suppression algorithm In this example, drawn for 3 genes for better understanding, we show the suppression of two attractor basins of the network, namely the states |001⟩ and |111⟩. The numbered steps on top refer to the phases defined in algorithm description, while the estimation of the basin size is omitted for better visualization of the attractor’s measurement routine. Specifically, step 1.2: apply a tailored −ϕ phase shift to the t=T qubits to mark undesired attractor basins, exploiting the forward $\hat{T}$ and reverse ${\hat{T}}^{†}$ time-evolution operators, responsible for implementing the Boolean network dynamics on the quantum device. Step 1.3: apply a layer of Hadamard gates to the t=0 qubits. Step 1.4: apply a ϕ phase shift to the t=0 qubits. Step 1.5: apply a layer of Hadamard gates to the t=0 qubits. The final measurement after the final time-evolution operator (step 2) will lead to the measurement of an attractor state not yet uncovered and suppressed.

In the following, we will implement the BFs, provided in text files formatted by the BoolNet R package,^18^ on quantum circuits using the ClassicalFunction compiler of the tweedledum package used by Qiskit.^30^ This way, we can generate quantum circuits implementing T state transitions of an n-component network using (T+1)·n qubits.^21^ As introduced in the previous section, the algorithm comprises two parts, which are to be carried out consecutively on each run. A comprehensive sketch of its structure is presented in Figure 5.

The first part consists of the development of a state initialization routine that initializes the system in the superposition of all states belonging to the attraction basins of attractors that have yet to be found. On the first run of the algorithm, this part trivially consists of a layer of Hadamard gates alone. A seminal paper by Liu and Ouyang^31^ theoretically demonstrated a modification of Grover’s algorithm that can delete a specific set of M marked states from a uniform superposition over all $N=2^{n}$ basis states. Here, we have taken inspiration from their result to develop an algorithm (and its circuit implementation) adapted to fit our purpose.

**Figure 6:**
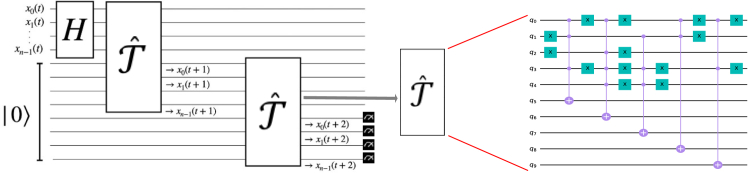
Schematic of a circuit implementing the time-evolution dynamics of a given Boolean network via the operator $\hat{T}$, which generates the single-step time evolution of the system The scheme on the left produces a superposition of all attractors, and the final measurements will lead to finding one of them. On the right is an example of what a time-evolution operator can look like for a given set of Boolean rules responsible for generating the network dynamics.

Our algorithm consists of five steps. The first step is to find out how many states we want to suppress from the superposition, after which we can write the operator ${\hat{S}}_{t}$ that performs the suppression of the selected states from a Hadamard state $|\psi \rangle ={\hat{H}}^{⊗n}|0\rangle_{n}$ as follows:

$${\hat{S}}_{t}|\psi \rangle =-{\hat{H}}^{⊗n}\hat{I_{0}}{\hat{H}}^{⊗n}\left({\hat{T}}_{t}^{†}\hat{I_{c}}{\hat{T}}_{t}\right)|\psi \rangle \text{,}$$

where each operator represents one of the other four steps required for our state initialization routine. Specifically, the $\hat{T}$ operator encodes the dynamical rules of the specific network under analysis and is responsible for evolving a given state to its next time step. An example of such an operator is given in Figure 6. We list and describe these 5 steps here and show our implementation on the quantum device.

• Step 1.1: estimate the number of states to be suppressed. Following the strategy used by Weidner et al.,^21^ we run the quantum counting algorithm^32^ once to obtain the number of states we want to erase from the total superposition (i.e., the number of states that belong to attractor basins already discovered). This parameter is used to estimate the angle ϕ used in steps 2 and 4. A different option, not pursued in this work, is to adapt the strategy developed by Boyer et al.^33^ for a Grover search with an unknown number of searched elements.
• Step 1.2: phase shift applied to all computational basis states not to be suppressed (not marked). This step is the most complex, as we want *not to mark* a set of states (all states belonging to one or more known attractors) that are not generally known. To do so, after an initial layer of Hadamard gates, we first implement the operator ${\hat{T}}_{t}$, which evolves our system in time until the system converges to the superposition of the attractors. At this point, by applying the operator $\hat{I_{c}}$,

$$\hat{I_{c}}=\hat{I}+\left(e^{i\phi }-1\right)\sum_{i\neq \tau }|i\rangle \langle i|\text{,}$$

we apply the phase shift to all attractors whose basins we do not want to suppress. To do this more efficiently, we instead mark with an opposite phase −ϕ the actual attractor(s) whose bases are to be suppressed since a global shift of +ϕ to all states (corresponding to the identity operator) would lead to the desired result. The angle ϕ will be specified later, and τ is the basis state (or set of basis states) to be suppressed. Introducing such tailored phase shifts, rather than the kickback method implemented in Grover’s amplification algorithm, is the key to optimizing the algorithm so that only one query is required for perfect suppression. Its derivation is shown in the work of Liu et al.^31^ The technical implementation of the phase-shift gates, which apply the desired phase shift to the required states, is instead a generalization of the algorithm developed by Fujiwara et al.,^34^ where instead of a π shift, we implement a more general ϕ shift. In the supplemental information, we include a short description of the implementation of such a routine. Finally, we propagate the phase shifts from the attractors throughout their entire basins by reversing the initial time evolution, accomplished by applying the operator ${\hat{T}}_{t}^{†}$ to the system. More details on the use of time-evolution operators can be found in Weidner et al.^21^^,^^23^
• Step 1.3: layer of Hadamard gates applied to all qubits, ${\hat{H}}^{⊗n}$.
• Step 1.4: conditional phase shift of $e^{-i\phi }$ applied to the $|0\rangle_{n}$ state and of $e^{i\pi }$ applied to all other states:

$$-\hat{I_{0}}=-\hat{I}-\left(e^{i\phi }-1\right)|0\rangle \langle 0|\text{,}$$

where the ϕ has to match that of step 1. Adopting a similar strategy as in step 1.2, we apply a global shift to the transformation, leading us to only apply a phase shift of $e^{+i\phi }$ applied to the $|0\rangle_{n}$ state.
• Step 1.5: layer of Hadamard gates applied to all qubits, ${\hat{H}}^{⊗n}$.

A single application of this routine will ensure the exact suppression of a set of M basis states from a uniform superposition of N states as long as the condition $\frac{M}{N}<\frac{3}{4}$ holds. More in general, the optimal number of iterations J this subroutine requires to exactly suppress M states is

$$J=\lceil \frac{\pi }{2\pi -4\beta }-\frac{1}{2}\rceil \text{,}$$

where ⌈x⌉ represent the ceiling function ceil (x) and $\beta =\arcsin\left(\sqrt{M/N}\right)$. Following this definition, we can now define the angle ϕ introduced above as

$$\phi =2\;\arcsin\left(\frac{\sin\left(\frac{\pi }{4J+2}\right)}{\cos\left(\beta \right)}\right)\text{.}$$

More theoretical insights about this procedure can be found in the paper of Liu et al.^31^

After the suppression of the states we are not interested in measuring from the initial uniform superposition, in the second part of the algorithm, we simply apply the time-evolution operator ${\hat{T}}_{t}$ again and then measure the system. The suppression performed in the first part of the algorithm ensures that we will now measure a different static or dynamic attractor than the ones we found before.

### Conclusions

This work introduces a novel method for quantum computation that provides the exact solution to the problem of attractor search in BNs, complementing a recent line of investigation addressing related problems via quantum computing techniques.^35^ Such a problem is strongly motivated by research in various fields, finding applications in theoretical computation, physics, and engineering research. Here, we introduce the problem as a way to study the behavior of GRNs. Indeed, BNs are often used to model the regulatory relationships between genes, and attractors in these networks correspond to stable gene expression patterns that can represent different cellular states, such as cell types or conditions (e.g., healthy vs. diseased states).

Our algorithm, developed by combining and evolving subroutines from different sources and further integrated with classical methods to enhance its stability and practicality, has resulted in what is, to the authors’ knowledge, a novel technique in the field of BN analysis, belonging to a series of seminal works that have recently brought the potential of quantum platforms to this field. We find that our algorithm is theoretically able to detect all attractors of any synchronous BN, even those with the smallest basins of attraction, with 100% accuracy, in a number of queries corresponding to the number of attractors present in the system itself. To the best of the authors’ knowledge, no algorithm in the literature currently achieves such a result. Moreover, runs simulating the results of state-of-the-art noisy quantum machines show that this method is stable, on relatively small example systems, against the errors induced by the current NISQ nature of quantum devices. This finding is promising for the development of future, more fault-tolerant quantum technologies. In addition, we would like to highlight how this method can be further improved (both in terms of efficiency and error tolerance) by integrating classical computational techniques, such as validating the true attractor nature of a state found as an attractor by the quantum algorithm, which can be performed in the range of seconds even on very large networks.

Finally, as an alternative to the gate-based circuit, we would like to mention the possibility of implementing such an algorithm on a quantum annealer. Such hardware is specialized for solving optimization problems in QUBO form, and there are already methods to implement the logical AND, OR, and NOT operations as constraints in this form. Thus, it may be possible to take advantage of the larger number of qubits available on quantum annealers (over 5,000 at the time of writing for D-Wave machines) compared to gate-based quantum computers (a maximum of 1,121 qubits on the IBM Condor processor). This may allow faster scaling toward the analysis of larger networks, although the overhead in the number of qubits required to encode the logical constraints and to perform error correction must first be established.

## Methods

Detailed methods can be found in the supplemental methods.

## Resource availability

### Lead contact

Requests for further information and resources should be directed to and will be fulfilled by the lead contact, Hans A. Kestler (hans.kestler@uni-ulm.de).

### Materials availability

Not applicable; see data and code availability.

### Data and code availability

The code for performing the analyses shown in this work as well as generating the resulting visualizations is available at https://github.com/sysbio-bioinf/QuantumAttractorSearch and has been archived at Zenodo.^36^

## Acknowledgments

H.A.K. acknowledges funding from the German Science Foundation (10.13039/501100001659DFG, SFB 1506, grant no. 450627322). J.A. acknowledges funding from the Center for Integrated Quantum Science and Technology (${\text{IQ}}^{\text{ST}}$) and the 10.13039/100008316Baden-Württemberg Foundation via the network program QT.BW.

## Author contributions

M.R. and F.M.W. contributed equally to the development of this work, while J.A. and H.A.K. conceptualized and supervised the project and contributed to the realization of this manuscript. All authors read and approved the final manuscript.

## Declaration of interests

The authors declare no competing interests.

## Supplemental information

Document S1. Figures S1–S4 and supplemental methods

Document S2. Article plus supplemental information
