# Supplementary material for: A novel quantum algorithm for efficient attractor search in gene regulatory networks
Source: Patterns (N Y). 2025 Jul 3;6(9):101295. doi: 10.1016/j.patter.2025.101295 (PMC12485557; doi:10.1016/j.patter.2025.101295)
Supplement: Document S1. Figures S1–S4 and supplemental methods [file mmc1.pdf]

Patterns, Volume *6*

Supplemental information

A novel quantum algorithm for efficient attractor search in gene regulatory networks

Mirko Rossini, Felix M. Weidner, Joachim Ankerhold, and Hans A. Kestler

Supplementary Material

S1. FOUR AGENTS TEST BOOLEAN NETWORK

The following table describes the logic rules that make up the updating scheme of the 4 agents test Boolean network used to demonstrate the effectiveness of the algorithm presented in this paper.

Time evolved agents	Logic rules for the updating scheme
$x_0(t+1)$	$x_0(t)$ & $x_3(t)$
$x_1(t+1)$	$x_0(t)$ & $x_1(t)$
$x_2(t+1)$	$x_2(t)$ & $x_3(t)$
$x_3(t+1)$	$x_1(t) \mid x_2(t)$

To show their implementation, assume we initiate the 4 agents in the state $x_0 = 0,\ x_1 = 1,\ x_2 = 0,\ x_3 = 1 \rightarrow 0101$. Applying the rules above lead us to the following updating scheme:

Time evolved agents	Logic rules for the updating scheme
$x_0(1)$	$x_0(0)$ & $x_3(0) \rightarrow 0$ & $1 \rightarrow \underline{0}$
$x_1(1)$	$x_0(0)$ & $x_1(0) \rightarrow 0$ & $1 \rightarrow \underline{0}$
$x_2(1)$	$x_2(0)$ & $x_3(0) \rightarrow 0$ & $1 \rightarrow \underline{0}$
$x_3(1)$	$x_1(0) \mid x_2(0) \rightarrow 1 \mid 0 \rightarrow \underline{1}$

leading to the state 0001. As can be seen in the figure below, a further application of the above mapping leads to the state 0000, which is one of the attractors of the system.

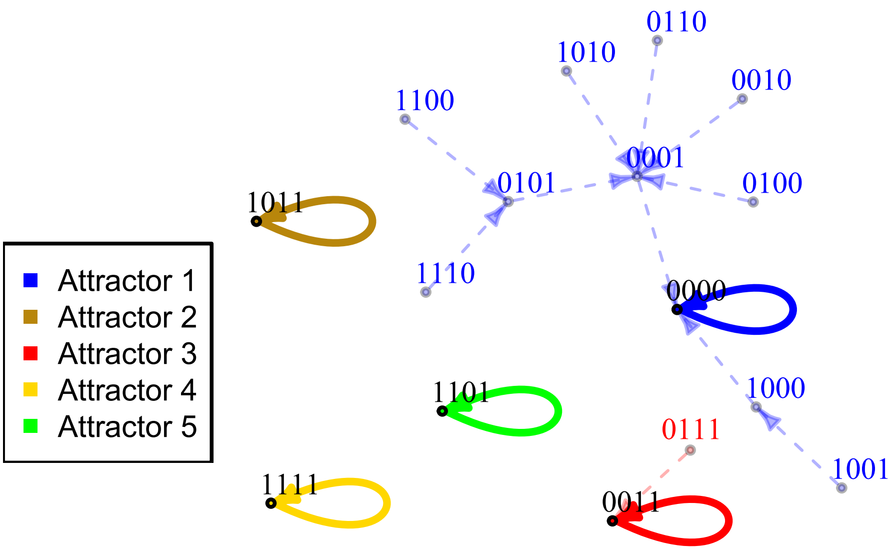

FIG. S1. : Schematic representation of the Boolean network generated by 4 interacting agents designed ad hoc as a test case.

S2. IMPLEMENTATION OF CONDITIONAL PHASE SHIFTS

We utilize this Grover-based deletion of states and combine it with the gate-based implementation of Boolean logic circuits presented by Weidner et al. [?] and described in detail in [?]. For this purpose, it is required to implement conditional phase shift operators $\hat{\Phi}_j^n$ which applies a phase $e^{i\phi}$ the j-th basis state in an n qubit system while leaving all others unchanged. Here, $j \in \{0, ..., 2^n - 1\}$, with j corresponding to the basis states $0 = |0...00\rangle, 1 = |0...01\rangle, 2 = |0...10\rangle$ and so on.

Mathematically, such phase shift is described by the matrix:

$$\hat{\Phi}_j^n = \begin{bmatrix} 1 & & & & \\ & 1 & & 0 & \\ & & \ddots & & \\ & & & e^{i\phi} & \\ & 0 & & & \ddots & \\ & & & & & 1 \end{bmatrix} \tag{1}$$

The phase ϕ for these operators is calculated based on the ratio of the number of marked basis states over the total amount of states $\frac{M}{N}$, $N = 2^n$, in order to completely suppress the target states. The value of M corresponds to the basin size of the already identified attractors and can be obtained by running a quantum counting circuit [? ?].

A gate-based implementation of such phase shift operators is described by Fujiwara and Hasegawa [?].

These phase shift operators were specified by Fujiwara and Hasegawa for a base case of $n = 2$ qubits as

$$C_0^2 = \hat{\Phi}_2^2 = \begin{bmatrix} 1 & 0 & 0 & 0 \\ 0 & 1 & 0 & 0 \\ 0 & 0 & e^{i\phi} & 0 \\ 0 & 0 & 0 & 1 \end{bmatrix}, \quad C_1^2 = \hat{\Phi}_3^2 = \begin{bmatrix} 1 & 0 & 0 & 0 \\ 0 & 1 & 0 & 0 \\ 0 & 0 & 1 & 0 \\ 0 & 0 & 0 & e^{i\phi} \end{bmatrix}. \tag{2}$$

Based on this, phases can be applied to the remaining two basis states by construction of the operators $\hat{\Phi}_1^2 = N_c \hat{\Phi}_3^2 N_c$ and $\hat{\Phi}_0^2 = N_c \hat{\Phi}_2^2 N_c$, where N_c indicates a layer of NOT gates to be applied to the qubits corresponding to the zeros of the basis state to shift. An example is provided in Fig.S2

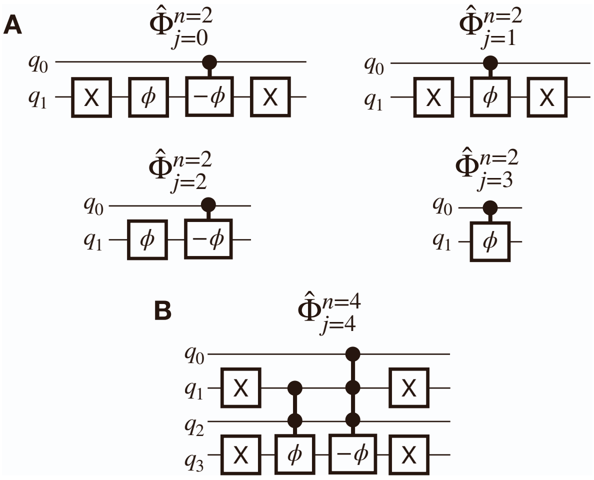

FIG. S2. Quantum circuits for the conditional state shifters specified by the corresponding values of n and j.

S3. SCALING OF THE REMAINING STATE SPACE AFTER REPEATED ITERATIONS

Up to $M/N = 3/4$ of the remaining state space can be suppressed in $j = 1$ iteration. Therefore, the fraction of basis states that can be suppressed in a space of size N starting from a uniform superposition over all N basis states in j iterations of the Grover operator is $1 - (\frac{1}{4})^j$.

The number of iterations J that have to be performed is calculated depending on the ratio of marked states in the state space M/N contained in the parameter β as ([?]):

$$\beta = \arcsin(\sqrt{M/N}) \tag{3}$$

$$J = \left\lceil \frac{\beta}{\pi - 2\beta} \right\rceil. \tag{4}$$

Based on this, the angle ϕ used in the phase shift operator is adapted to a value of

$$\phi = 2 \cdot \arcsin\left(\frac{\sin(\pi/(4J+2))}{\cos(\beta)}\right) \tag{5}$$

in every iteration of the Grover operator.

S4. EXAMPLE RUN WITH CYCLIC ATTRACTORS

Here we demonstrate the methodology for studying the attractors of a network in the presence of possible cyclic attractors. The rules of the network are given in the table below and the corresponding state transition graph is shown in Fig.S3.

Time evolved agents	Logic rules for the updating scheme
$x_0(t+1)$	$x_0(t)$ & $x_2(t)$
$x_1(t+1)$	$\neg\, x_0(t)$ & $x_1(t)$
$x_2(t+1)$	$\neg\, x_1(t)$
$x_3(t+1)$	$\neg\, x_2(t)$ & $\neg\, x_3(t)$

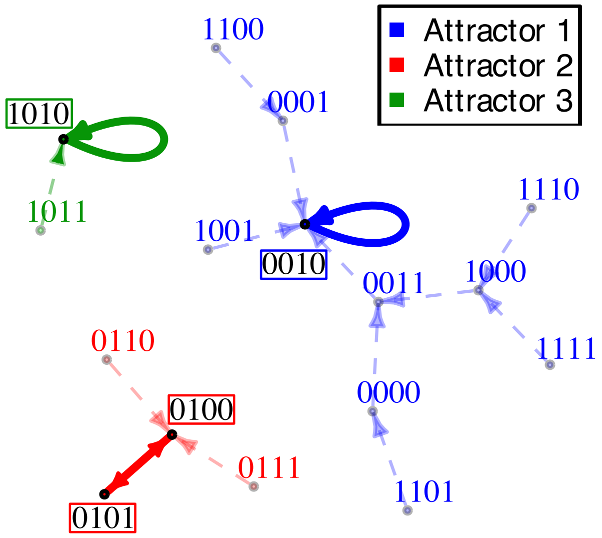

FIG. S3. STG for the Boolean network described by the rules in the table above. It presents two static attractors and one cyclic attractor composed of the two states 0100 and 0101.

Applying the time evolution operator to this network will cause it to converge to a final superposition of states where all static attractors and all states belonging to cyclic attractors are present, as shown in the first histogram of Fig.S4 (1st run).

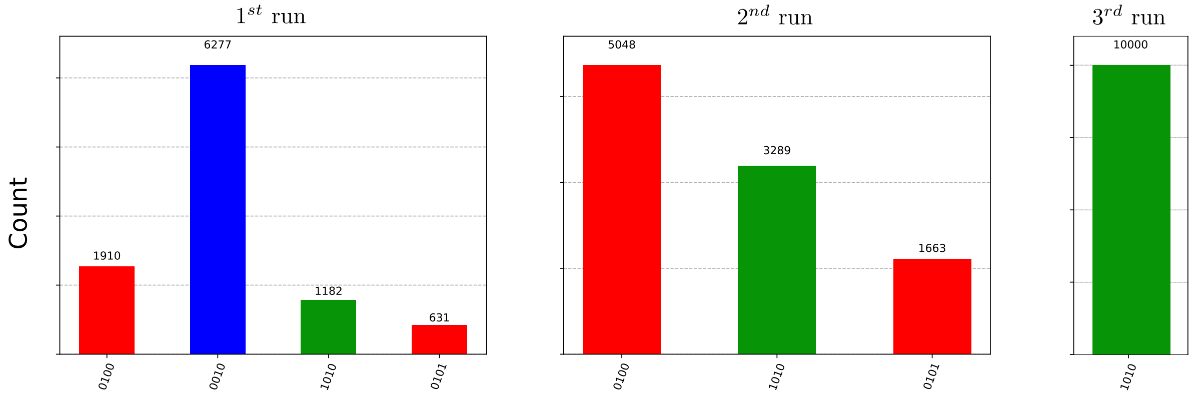

FIG. S4. Example run of the algorithm on the network. In the first run, no attractors are suppressed, and state 0010—later identified as a static attractor—is measured. Suppressing its basin reveals a new distribution in the second run, where state 0100 is measured and ten identified as part of a cyclic attractor composed of 0100 and 0101. In the third run, all previously discovered attractor states are suppressed, leading to the final measurement of the static attractor 1010.

Fig.S4 shows an example run of our algorithm on this network. After the first run, where no attractors are suppressed, we suppose to measure state 0010, which is the most likely attractor to be

measured. A quick classical evaluation shows that this is a static attractor. Suppressing its attractor basin leads to the next distribution of uncovered attractors in the 2nd run. We now suppose to measure the state 0100, which is one of the two attractors belonging to the cyclic attractor. Once again we classically investigate the nature of this attractor, revealing its cyclic nature and all the states of which it is composed – 0100 and 0101. For the 3rd we proceed to mark all the states belonging to the cyclic and static attractors from the previous runs (0010, 0100 and 0101), which gives us a final 100% probability to measure the last attractor, 1010, whose static nature can once again be easily revealed classically.
